# The effect of virtual reality on anxiety, stress, and hemodynamic parameters during cesarean section: A randomized controlled clinical trial

**DOI:** 10.15537/smj.2022.43.4.20210921

**Published:** 2022-04

**Authors:** Sultan A. Almedhesh, Wafaa T. Elgzar, Heba A. Ibrahim, Hiba A. Osman

**Affiliations:** *From the Department of Pediatric And Pediatric Cardiologist (Almedhesh), College of Medicine; from the Department of Maternity and Childhood Nursing (Elgzar, Ibrahim), Nursing College Najran University; and from the Department of Obstetrics and Gynecology (Osman), Maternity and Children Hospital, Najran, Kingdom of Saudi Arabia.*

**Keywords:** anxiety, caesarian section, hemodynamic, stress

## Abstract

**Objectives:**

To investigate the effect of virtual reality (VR) on anxiety, stress, and hemodynamic parameters during cesarean section (CS).

**Methods:**

This is a randomized controlled clinical trial conducted at the operating theatre / Maternal and Children Hospital, Najran, Saudi Arabia from February to October 2021. The study comprised a random sample of 351(176 study and 175 control) low-risk pregnant women undergoing elective CS with regional anesthesia. Data collection was carried out using 5 instruments. Basic and clinical data sheet, maternal hemodynamic parameters assessment sheet, brief measure of preoperative emotional stress, a novel visual facial anxiety scale, and maternal satisfaction scale. Virtual reality group exposed to 3D natural videos associated with calm Quran or music voices via phone using VR glasses immediately after anesthesia until completion of skin suture. The control group left for routine hospital care.

**Results:**

The VR group showed significantly lower stress and anxiety levels immediately after skin suture and 2h postoperative (*p*=0.000). Maternal satisfaction 2 hours after CS showed that 58% of the VR group were completely satisfied compared to 11.3% of the control group (FET=135.359 *p*=0.000). Virtual reality have an impact on hemodynamic parameters at some time points while peripheral oxygen saturation did not differ significantly (*p*>0.05).

**Conclusion:**

Virtual reality significantly reduced anxiety and stress among women undergoing CS under regional anesthesia. Virtual reality may be added to the routine intraoperative techniques that help induce patient relaxation and increase satisfaction.

**C**esarean section (CS) prevalence has significantly increased in developed and developing countries.^1,2^ Preceding reports from various regions of the Kingdom of Saudi Arabia (KSA) have shown an alarming increase in the CS rate of more than 80% from 10.6% in 1997 to 19.1% in 2006.^3^ Furthermore, in 2018 the CS rate significantly increased to 27.5%, according to a recent study carried out in the King Abdulaziz Medical City, Jeddah, KSA^4^ which exceeds the acceptable and recommended rate (10-15%) by the World Health Organization (WHO).^5^ Hence, it is the most popular abdominal surgery and one of the most popular operations in general.

Cesarean section is mainly performed using regional anesthesia, without preoperative sedatives, to facilitate the mother’s conscious birth experience, reduce the need for neonatal resuscitation, and promote skin-to-skin contact immediately after birth between the mother and newborn.^6^

Although CS is considered a relatively popular method of childbirth, more than 80% of women experience a significant level of anxiety and stress before and during surgery, which leads to physiological and psychological risks. The previous study has indicated that CS-related anxiety and stress are associated with an increased risk of postpartum depression.^7^ Furthermore, excessive stress and anxiety before and during surgery may increase anesthesia related complications during the operation, increase postoperative analgesic requirement, prolonged recovery, and delayed lactation.^8^ It is essential to reduce CS-related stress and anxiety because lower preoperative/intraoperative stress and anxiety lead to better maternal satisfaction and a more positive birth experience.^9^

In light of the limited pharmacological choices for pregnant women during CS, there is a need for alternative and low-risk options that positively affect intraoperative anxiety and stress, especially if performed under regional anesthesia.^8^ An alternative non-pharmacological stress reduction method is the use of virtual reality (VR). VR is a computer-assisted technology that simulates a real-life environment by integrating 3D virtual objects to create a completely virtual environment surrounding the user’s eyes to replace the natural environment.^10^ The VR can be designed to be an interacting and emotionally engaging environment that can stimulate emotionally related hormones. In stressful situations as CS, VR can generate a relaxation state that improves the surgery outcomes.^11^

The application of VR varies widely according to the purpose of its use. It is widely used for medical education, pain relief, posttraumatic stress,gait rehabilitation in Parkinson’s disease patients, anxiety, and stress.^10-15^ The promising VR effect makes it suitable for non-pharmacological stress reduction methods in different stressful situations during surgical procedures. In addition, it needs no or little control or preparation on the patient and the health care practitioner. Therefore, the current study investigates the effect of VR on anxiety, stress, and hemodynamic parameters during CS.

## Methods

A randomized controlled clinical trial was followed in this study. It was conducted at the operating theatre (OT) / Maternal and Children Hospital (MCH), Najran, Saudi Arabia. It was carried out from February to October 2021. The trial was registered in the Iranian Registry of Clinical Trial with the number IRCT20210131050192N2. The effect of an independent variable (virtual reality) on dependent variables (maternal anxiety, stress, satisfaction, and hemodynamic parameters) were investigated. The study comprised a random sample of 351 low-risk pregnant women undergoing elective CS with regional anesthesia. Inclusion criteria were parturient with normal vision and hearing abilities, no history of generalized anxiety disorder or mental illness, free from serious obstetrics complications (according to the obstetrician evaluation), no increased intraoperative risk (such as, placental disturbance) that identified in the preoperative period and accepted to participate on the study. Any woman who developed intraoperative complications was excluded from the study.

The sample size was calculated using Yamane’s formula to make the maximum representation of the study population. According to the hospital statistical center, the sample size was calculated based on the number of CS performed at MCH hospital from January to December 2020, which was 2721 cases. Based on Yamane’s 16 formula, the sample size was calculated as the following:


$$\text{n}=\frac{\text{N}}{1+\text{N}(\text{e})2}=\text{n}=\frac{2721}{1+2721(0.05)2}=348.73$$

**Figure 1:**
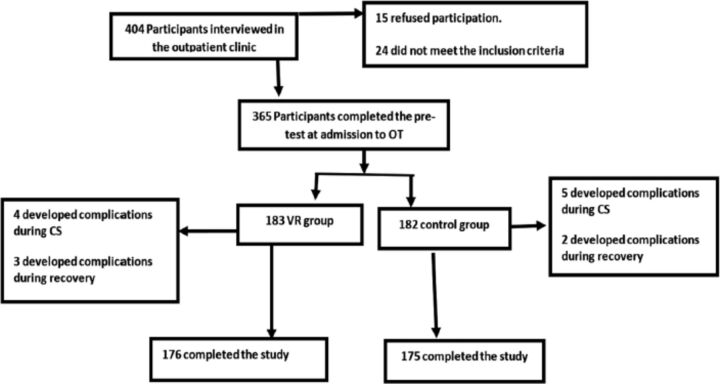
- Participant’s flowchart. CS: cesarean section, OT: operating theater

Where: n=sample size, N=total population number (2721), e=margin error (0.05). The participants were included in the study according to the following followchart (Figure 1).

A total of 351 participants were included in the study. Computer-assisted randomization was carried out to randomly assign 176 participants for the intervention group and 175 participants for the control group.

Data collection was carried out using 5 instruments after reviewing the related research on PUBMED website. Instrument I - basic and clinical datasheet: The researchers developed it to collect basic data such as age, weight, height, gestational age, gravity, parity. It also includes basic CS data, such as the number of previous CS, duration of current CS, amount of intravenous fluids, and blood loss during CS. Instrument II - Maternal hemodynamic parameters assessment sheet (objective parameters): This part was used to register the heart rate (HR), systolic blood pressure (SBP) and diastolic blood pressure (DBP), and peripheral oxygen saturation (SpO_2_) at patient admission OT, immediately after anesthesia, skin incision, delivery of the baby, skin suture and 2 hours (h) postoperative. Instrument III: - The brief measure of emotional preoperative stress (B-MEPS): The brief version of B-MEPS was modified by Wolmeister et al^17^ to measure preoperative emotional stress. The tool comprises 12 items; 3 of them are rated on a 4-point Likert scale, 6 are rated on a 3-point Likert scale, and the remaining 3 have 2 answers. The high score indicates high stress, and the total scale score ranged from 12-36. Instrument: IV: A novel visual facial anxiety scale (NVFAS): Cao et al^18^ developed a self-reported scale to assess acute (state) anxiety during clinical practices. It is composed of 11 faces that asses different degrees of anxiety from (0) no anxiety to (10) the highest anxiety level. Instrument V: The Birth Satisfaction Scale-Revised (BSS-R): It is composed of 10 statements rated on a 5-points Likert scale ranging from strongly disagree (1) to strongly agree (5). The patient was considered to be completely unsatisfied (10-18), unsatisfied (19-26), neutral (27-34), satisfied (35-42), and completely satisfied (43-50) based on her total satisfaction score.^19^

An official permission was obtained from the deanship of scientific research at Najran University, Najran, KSA. Another official approval was obtain from the health affairs administration at Najran. Ethical approval number (IRB Log Number 2021-29). After approval of the health affairs to conduct the study, official permission was obtain from the MCH director. Written informed consent was taken from each participant at the beginning of the study. The participants had the right to refuse participation or withdraw at any time. Furthermore, all the participants’ data were treated confidentially.

**Figure 2:**
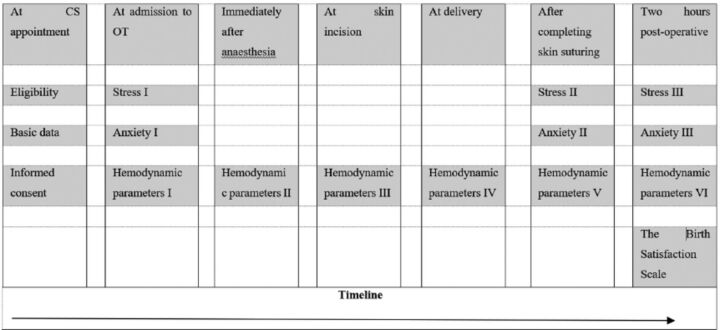
- Filed workflow chart.

The instruments were translated to the Arabic language and tested for content validity by a jury of 5 nursing experts and reliability by Cronbach’s alpha test. A pilot study was performed on 10% of the participants (excluded from the main sample) to ascertain the instruments’ clarity and applicability; then, the necessary changes were undertaken. Each woman was interviewed individually at the outpatient clinic at the CS appointment time to ensure eligibility for the study, collect basic data, and take informed consent to participate. On admission to the OT, all women in the VR and control group were evaluated for stress, anxiety, and hemodynamic parameters using the pre-described instruments (Figure 2).

For the VR group, the VR glasses were applied after regional anesthesia and during the whole operation time till completing skin suturing. The used VR glasses is Oculus Rift S PC-Powered VR Headset made in China. Every participant has to choose between listening to a Holy Quran in a sweet voice with natural landscapes and spiritual places or listening to calm, relaxing music with beautiful landscapes during surgery. Hemodynamic parameters were assessed before the intervention, immediately after anesthesia, skin incision, delivery of the baby, skin suture, and 2h postoperative. Anxiety was reassessed after completing skin suture and 2h postoperative. Brief measure of emotional preoperative stress and NVFAS were reassessed after completing skin sutures and 2 hours postoperative. In addition, patient satisfaction was assessed using a BSS-R 2h postoperative.

For the control group, they were left for routine hospital care. The same measurement timing for B-MEPS and NVFAS, hemodynamic parameters, and BSS-R were followed.

Data analysis were performed using the SPSS, version 23 (IBM Corp., Armonk, N.Y., USA)’. Before sending data to the statistician, a code was given to each group to ensure blind data analysis. To control the environmental confounding factors that may contaminate the results, only elective CS performed during the morning shift were included. In addition, the basic clinical data among the 2 groups were compared using an independent sample T-test to ensure the homogeneity of the study participants. To analyze the subjective variables of stress and anxiety at the day of CS 2X3 mixed-factorial ANOVAs between subjects’ factor group (VR versus control) and within-subject factor measurement times (at admission to OT, after completing skin suturing, and 2h postoperative) was performed. For analyzing the objective variables (SBP, DBP, HR, and SpO_2_), 2x6 mixed-factorial ANOVAs were utilized between-subjects factor group (VR versus control) and within-subject factor measurement times (at admission to OT, immediately after anesthesia, at skin incision, at delivery, after completing skin suture, and 2h postoperative). The adjusted Bonferroni was used to compare between VR and control group at each time point. The 2 groups’ maternal satisfaction was compared using Fisher exact test. Correction of the degree of freedom according to Greenhouse-Geisser was carried out if the sphericity was not assumed. The results were assessed within the 95% confidence interval (CI), and the *p*-value was considered significant at <0.05.

## Results

A total of 404 participants were screened at CS appointments; among them, 351 completed the study (Figure 1). Analysis of the basic and clinical data showed homogeneity of the 2 groups (*p*>0.05). The mean age was 31.20 years in the VR group compared to 32.28 years in the control group. Body mass index was 32.65±4.47 among the VR group Compared to 33.61±5.89 in the control group. The mean gravidity, parity, and gestational age were 4.25±2.36, 3.63±2.49 and 38.53± 1.12 among the VR group compared to 4.68±2.20, 3.86±2.02 and 38.43±1.01 among the control group, respectively. The previous CS mean was 2.28±1.70 among the VR group compared to 2.59±1.73 in the control group. In addition, the mean duration of CS was 46.88 and 48.16 minutes among VR and control groups, respectively. The mean amount of blood loss among the VR group was 601.13 ml compared to 585.42 ml in the control group. Finally, the mean of received IV fluid was 1226.13 and 1190.28 ml among VR and control groups, respectively.

### Mixed factorial ANOVAs for subjective parameters (B-MEPS and NVFAS) (Table 1)

For B-MEPS, the 2×3 mixed factorial ANOVAs showed significant differences between the VR and control group [F(1)=109.511, *p*=0.000] with significant mean effect for time factor [F(1.438)=1284.153, *p*=0.000] and significant time group interaction [F(1.438)=61.307, *p*=0.000]. An adjusted Bonferroni comparison throughout each time point showed no significant difference between the 2 groups regarding the stress level at admission to OT [F(1)=1.784, *p*=0.183, mean differences= 0.427, 95% CI for d= -1.056, 0.202], while, after completion of skin suturing the VR group showed significantly lower stress level [F=(1)173.579, *p*=0.000, mean differences= -3.109, 95% CI for d= -3.57,-2.646]. In addition, 2h post-operative the VR group had significantly lower stress mean score [F(1)=273.635, *p*=0.000, mean differences= -2.65, 95% CI for d= -2.96,-2.33]. Regarding the NVFAS, mixed factorial ANOVAs showed significant differences between VR and control group in relation to time [F(1.898)=1454.845, *p*=0.000], group [F(1.898)=292.192, *p*=0.000] and for time group interaction [F(1.898)=396.826, *p*=0.000]. The adjusted Bonferroni comparison between the 2 groups showed no significant difference in the anxiety levels at admission to OT [F(1)=1.791, *p*=0.182, mean differences= -0.126, CI 95% for d= -0.311, 0.059]. After skin suturing, VR group showed significantly lower anxiety level compared with control group [F(1)=330.417, *p*=0.000, mean differences= -2.115, CI 95% for d= -2.344, -1.886]. Again, 2h post-operative the VR group had significantly lower anxiety level [F(1)=580.417, *p*=0.000, mean differences= -2.406, CI 95% for d= -2.602, -2.209](Table 1).

**Table 1:** - Mixed factorial ANOVAs for subjective parameters (B-MEPS and NVFAS).

Group	Admission to OT (Mean±SD)	After skin suturing (Mean±SD)	2 hours postoperative (Mean±SD)	F for time (*P*-value)	F for group (*P*-value)	F for time* group interaction (*P*-value)
B-MEPS				1284.153 (**0.000**)	109.511 (**0.000**)	61.307 (**0.000**)
VR group	30.74±3.13	14.21±2.16	12.76±1.27			
Control group	31.17±2.84	17.32±2.25	15.41±1.69			
F(P-value)	1.784(0.183)	173.579 (**0.000**)	273.635 (**0.000**)			
NVFAS				1454.845 (**0.000**)	292.192 (**0.000**)	396.826 (**0.000**)
VR group	9.07±0.88	2.09±1.19	1.00±0.95			
Control group	9.20±0.87	4.21±0.97	3.41±0.91			
F(P-value)	1.791(0.182)	330.417(0.000)	580.417(**0.000**)			

Bold values indicate a significant difference between groups (*p*<0.05). OT: operating theatre, VR: virtual reality, B-MEPS: brief measure of emotional preoperative stress, NVFAS: novel visual facial anxiety scale

### Mixed factorial ANOVAs for objective parameters (maternal hemodynamic variables) (Table 2)

The SBP changed significantly with time factor based on the result of 2×6 mixed factorial ANOVAs [F(3.626)=86.957, *p*=0.000], while, no significant differences for group factor and group time interaction [F(3.626)=2.217, *p*=0.137 and F(3.626)=0.672, *p*=0.597], respectively. The adjusted Bonferroni comparison between the 2 groups showed no significant difference in the SBP at any time point except at delivery of the baby where the VR group had higher SBP compared to the control group [F(1)=5.331, *p*=0.022, mean differences=3.891, CI 95% for d=0.576, 7.205]. The DBP changed significantly with time and group factor [F(4.017)=145.611, *p*=0.000 and F(1)=8.678, *p*=0.003], respectively. No group time interaction was observed [F(4.017)=1.809, *p*=0.124]. The adjusted Bonferroni comparison between the 2 groups showed no significant difference in the DBP at any time point except at delivery and after skin suture [F(1)=14.211, *p*=0.000, mean differences= 5.248, CI 95% for d=2.510, 7.986 and F(1)=4.413, *p*=0.036, mean differences= 2.227, CI 95% for d= 0.142, 4.313], respectively. The HR changed significantly with time and group time interaction [F(2.821)=65.847, *p*=0.000 and F(1)=3.254, *p*=0.024], respectively. No significant difference in group factor found [F(2.821)=1.854, *p*=0.174]. The adjusted Bonferroni comparison between the 2 groups showed no significant difference in the HR at any time point except after skin suture and 2h postoperative [F(1)=13.166, *p*=0.000, mean differences= -4.440, CI 95% for d = -6.847, -2.033 and F(1)=9.145, *p*=0.003, mean differences= -2.771, CI 95% for d= -4.574, -0.969], respectively. The SpO_2_ changed significantly with the time factor [F(3.643)=29.329, *p*=0.000]. There is no significant difference in group factor or time group interaction were recorded [F(1)=2.389, *p*=0.123 and F(3.643)=1.010, *p*=0.397], respectively. The adjusted Bonferroni comparison between the 2 groups showed no significant difference in the SpO_2_ at any time points (Table 2)(Figure 4).

**Table 2:** - Mixed factorial ANOVAs for objective parameters (maternal hemodynamic variables).

Group	At admission to OT (Mean±SD)	Immediately after anesthesia (Mean±SD)	At skin incision (Mean±SD)	At delivery (Mean±SD)	After skin suturing (Mean±SD)	2 hours postoperative (Mean±SD)	F for time (*P*-value)	F for group (*P*-value)	F for time* group interaction (*P*-value)
SBP							86.957 (**0.000**)	2.217 (0.137)	0.672 (0.597)
VR group (n=176)	129.89±19.44	114.98±15.47	112.00±21.22	115.08±13.84	113.64±13.34	117.54±10.27			
Control group (n=175)	128.57±16.04	113.46±22.57	111.50±17.60	111.18±17.52	112.26±12.69	115.51±11.27			
F(P-value)	0.485(0.486)	0.538(0.464)	0.057(0.811)	5.331(**0.022**)	0.984(0.322)	3.094(0.079)			
DBP							145.611 (**0.000**)	8.678 (**0.003**)	1.809 (0.124)
VR group (n=176)	76.21±10.19	73.77±13.95	62.71±13.35	62.51±9.31	65.25±10.90	71.01±7.97			
Control group(n=175)	74.58±12.31	71.85±18.23	60.37±9.21	57.26±15.93	63.02±8.83	69.68±7.80			
F(P-value)	1.819(0.178)	1.229(0.268)	3.644(0.057)	14.211(**0.000**)	4.413(**0.036**)	2.496(0.115)			
HR							65.847 (**0.000**)	1.854 (0.174)	3.254 (0.024)
VR group (n=176)	101.15±20.54	97.95±18.69	91.46±22.05	90.46±17.27	87.15±12.44	87.08±10.22			
Control group (n=175)	100.46±17.65	96.88±16.28	93.89±11.88	92.68±12.30	91.59±10.38	89.85±6.52			
F(P-value)	0.114(0.736)	0.326(0.568)	1.642(0.201)	1.910(0.168)	13.166(0.000)	9.145(0.003)			
SpO_2_							29.329 (**0.000**)	2.389 (0.123)	1.010 (0.397)
VR group (n=176)	98.58±1.01	98.47±1.11	98.38±1.42	98.21±1.50	98.40±1.33	99.02±0.77			
Control group (n=175)	98.62±1.40	98.29±1.38	98.21±1.16	97.92±1.75	98.33±1.07	98.90±0.70			
F(P-value)	0.110(0.740)	1.805(0.180)	1.475(0.225)	2.875(0.091)	0.360(0.549)	2.096(0.149)			

Bold values indicate a significant difference between VR and control groups (*p*<0.05). VR: virtual reality, OT: operating theatre, VR: virtual reality, SBP.: systolic blood pressure, DBP: diastolic blood pressure, SpO_2_: peripheral oxygen saturation, HR: heart rate

**Figure 3:**
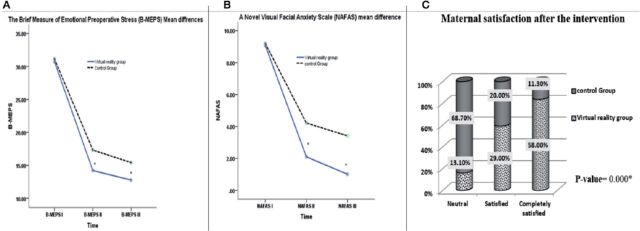
- The subjective parameters (B-MEPS [emotional preoperative stress], novel visual facial anxiety scale [NVFAS], and Birth Satisfaction Scale-Revised [BSS-R] among the 2 groups). **A**) For B-MEPS score the virtual reality (VR) group showed significantly lower stress levels immediately after skin suture and 2hours (h) postoprative compared to the control group. **B**) Regarding the NVFAS, the score decreased significantly in the VR group compared to the control group immediately after skin suture and 2h postoprative. **C**) The BSS-R 2h after CS showed that 58% of the VR group were completely satisfied compared to 11.3% of the control group with statistically significant difference (FET= 135. 359 *p*-value= 0.000)*p<0.05.

**Figure 4:**
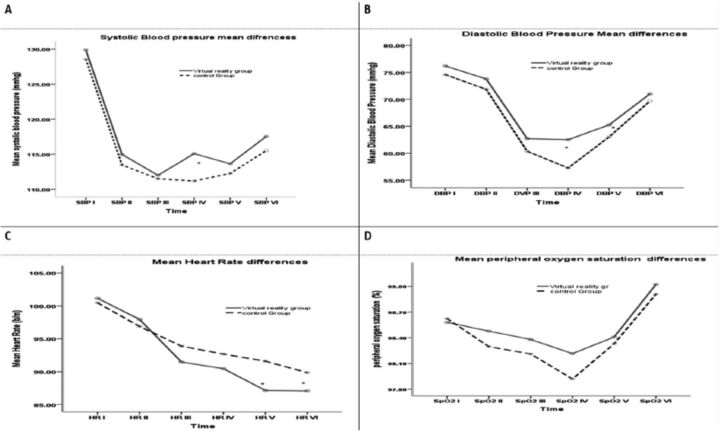
- The objective variables (maternal hemodynamic parameters among the 2 groups. **A)** The systolic blood pressure at the delivery time was significantly higher in the virtual reality [VR] group than the control group. **B)** The VR group showed significant increase in diastolic blood pressure at delivery and after skin suture. **C)** HR was decreased significantly in the VR group than the control group after skin suture and 2 hours postoperative. **D)** SpO_2_ did not differ significantly among VR and control groups at any time point. *p<0.05

## Discussion

Managing stress and anxiety associated with surgical interventions is a complex and challenging process. Incorporating non-pharmacological strategies has been one of the priorities of medical care reform in the past decades. After searching international data base, this study used an experimental design to determine the effectiveness of VR as a non-pharmacological approach in reducing stress and anxiety during CS, and with the assessment of subjective and objective parameters. In the current study, although patients in both groups demonstrated lower stress and anxiety scores across 2-time points (immediately after skin suture and 2h postoperative), results revealed that patients in the VR group obtained significantly lower stress and anxiety scores when compared to the control group. The results illustrate the effectiveness of VR technologies in reducing the stress and anxiety levels among patients undergoing CS using regional anesthesia. The current results suggested that the VR technique may generate positive feelings and mood improvements to decrease the patient’s anxiety before, during and after invasive procedures. In addition, VR glasses reduce the exposure of patients undergoing regional anesthesia to visual and auditory stimuli inside the OT and generate distractions that help to reduce stress and anxiety.

These findings are in accordance with a recent randomized controlled trial carriedout by Turan et al.^20^ The researchers indicated that VR was effective in reducing anxiety levels during surgical intervention under spinal anesthesia.^20^ Also, Frey et al^21^ reported that the anxiety score was significantly reduced in the VR group compared to the control group during unmedicated contractions in the 1st stage of labour. A systematic review of 23 studies by Ioannou et al^22^ found that VR effectively decreases symptoms of stress and anxiety in various contexts and diseases.A similar positive effect of VR technology in reducing stress and anxiety in other medical procedures have been reported by prior studies.^23,24^ In addition, VR distraction has also been shown to control stress and anxiety in patients undergoing dental treatment.^25-28^ Besides, VR technology is most frequently used in the management of anxiety disorders and other psychiatric diseases.^29,30^

On the contrary, Walker et al^31^ & Glennon et al^32^ reported no significant difference between the VR and control groups regarding anxiety scores among patients undergoing cystoscopy and bone marrow aspiration, respectively. The difference may be attributed to the different technological factors and the audiovisual material provided through the VR glasses, which may improve the degree of VR distraction to reduce stress and anxiety.

Nowadays, data collection on patient’s satisfaction is significant for assessing and improving the quality of medical care; therefore, the maternal satisfaction assessment took place in the current study. The present study results demonstrated that maternal satisfaction regarding the overall delivery process was significantly higher in the VR group than in the control group. More than half of the patients were completely satisfied in the VR group compared to around one-tenth of the control group. This result is in line with Alaterre et al,^33^ they reported that the satisfaction scores in the VR group were significantly higher than those of the control group in patients undergoing regional anesthesia. Moreover, Dumoulin et al^34^ found that children satisfaction concerning needle-related procedures was significantly higher in the VR group than TV group. Also, Tharion et al^35^ stated that the postoperative satisfaction score in the VR group was significantly higher than the midazolam group.

Regarding the hemodynamic parameters, the present study results showed no significant difference in the SBP at any time point except at delivery, where the VR group had higher SBP compared to the control group. The DBP does not change significantly among the 2 groups at any time point except at delivery and after skin suture. Furthermore, HR decreased significantly among the VR group compared to the control group after skin suture and 2h postoperative. Although the SpO_2_ changed significantly with the time factor, there is no significant difference in the 2 groups at any time point.

In line with the current study, Baytar and Bollucuoğlu conducted a quasi-experimental study to investigate the effect of VR glasses on preoperative anxiety before septorhinoplasty. They illustrated that HR significantly decreased after VR glasses use, while SpO_2_ did not differ significantly.^36^ Also, Tharion et al^35^ explored the effectiveness of VR on reducing anxiety in patients undergoing surgery under spinal anesthesia. They reported that VR did not significantly affect SpO_2_ or the other hemodynamic parameters except for respiratory rate. However, all the hemodynamic parameters were stable among both groups throughout the surgery. In addition, Sahin et al^37^ stated that VR utilization during surgery could help hemodynamic stabilization due to the stress reduction mechanism. The benefits of VR in pain reduction and physiological parameters among non-medicated labouring women was examined by Frey et al^21^ and found that VR significantly reduced HR and pain. Also, Hua et al^38^ reported that VR significantly decreased heart rate among children during dressing on chronic lower limb wounds. They added that SpO_2_ did not differ significantly among VR and control groups.

On the contrary to the present study, Baytar and Bollucuoğlu reported that VR significantly decreased SBP and DBP among their participants. The differences between the current study results and that of Baytar and Bollucuoğlu may be attributed to the different situations of data collection.^36^ The present study data were collected in OT, and the patient received regional anesthesia, which is known to be associated with hypotension and increasing HR.^39^ Therefore, stabilizing the patient’s hemodynamic state requires a slight elevation of SBP and DBP. Baytar and Bollucuoğlu^36^ collected their data before operation inside the patient room; therefore, the relaxation induced by using VR leads to a reduction in SBP and DBP, which were already elevated due to the physiologic mechanism of preoperative stress and anxiety.

### Study limitations and strengths

Blindness cannot be applied in this study at the data collection because of the procedure nature. There are some contributing factors to stress and anxiety that we could not control, such as religion, education, period of waiting before the operation, social support system and previous experience with OT. Both primiparous and multiparous women were included in the current study due to limited number of elective CS for primiparous. Strengths of this study include enough sample size (351) and repeated measurement at different time points. Also, we used standardized tools for data collection.

In conclusion, VR significantly reduced anxiety and stress among women undergoing CS under regional anesthesia. This safe, cheap, harmless, and easy to use method of stress reduction has a positive impact on some hemodynamic parameters and significantly increased patients’ satisfaction. Virtual reality may be added to the routine intraoperative techniques that help to induce patient relaxation and help to increase satisfaction. However, numerous studies are needed to confirm the benefit of VR intraoperative for primiparous women.

## References

[1] Verma V , Vishwakarma RK , Nath DC , Khan HTA , Prakash R , Abid O. Prevalence and determinants of caesarean section in South and South-East Asian women. PLoS One 2020; 15: e0229906. 32163440 10.1371/journal.pone.0229906 PMC7067459

[2] Betran AP , Torloni MR , Zhang J , Ye J , Mikolajczyk R , Deneux-Tharaux C , Oladapo OT , Souza JP , Tunçalp Ö , Vogel JP , Gülmezoglu AM. What is the optimal rate of caesarean section at population level? A systematic review of ecologic studies. Reprod Health 2015; 12: 57. 26093498 10.1186/s12978-015-0043-6 PMC4496821

[3] Ba’aqeel HS. Cesarean delivery rates in Saudi Arabia: a ten-year review. Ann Saudi Med 2009; 29: 179–183. 19448379 10.4103/0256-4947.51773 PMC2813649

[4] Alsulami SM , Ashmawi MT , Jarwan RO , Malli IA , Albar SK , Al-Jifree HM. The Rates of Cesarean Section Deliveries According to Robson Classification System During the Year of 2018 Among Patients in King Abdul-Aziz Medical City, Jeddah, Saudi Arabia. Cureus 2020; 12: e11529. 33354473 10.7759/cureus.11529 PMC7746316

[5] Norum J , Svee TE. Caesarean section rates and activity-based funding in Northern Norway: a model-based study using the World Health Organization’s recommendation. Obstet Gynecol Int 2018; 2018: 6764258. 30116268 10.1155/2018/6764258 PMC6079324

[6] Elsaharty A , McConachie I Skin to skin: A modern approach to Caesarean delivery. J Obstet Anaesth Crit Care 2017; 7: 13.

[7] Räisänen S , Lehto SM , Nielsen HS , Gissler M , Kramer MR , Heinonen S. Fear of childbirth predicts postpartum depression: a population-based analysis of 511 422 singleton births in Finland. BMJ Open 2013; 3: e004047. 10.1136/bmjopen-2013-004047 PMC3845069 24293208

[8] Hepp P , Hagenbeck C , Gilles J , Wolf OT , Goertz W , Janni W et al. Effects of music intervention during caesarean delivery on anxiety and stress of the mother a controlled, randomized study. BMC Pregnancy Childbirth 2018; 18: 435. 30390639 10.1186/s12884-018-2069-6 PMC6215648

[9] Noben L , Goossens SMTA , Truijens SEM , van Berckel MMG , Perquin CW , Slooter GD et al. A virtual reality video to improve information provision and reduce anxiety before cesarean delivery: randomized controlled trial. JMIR Ment Health 2019; 6: e15872. 31850850 10.2196/15872 PMC6939281

[10] Huang TK , Yang CH , Hsieh YH , Wang JC , Hung CC. Augmented reality (AR) and virtual reality (VR) applied in dentistry. Kaohsiung J Med Sci 2018; 34: 243–248. 29655414 10.1016/j.kjms.2018.01.009 PMC11915632

[11] Kothgassner OD , Goreis A , Kafka JX , Van Eickels RL , Plener PL , Felnhofer A. Virtual reality exposure therapy for posttraumatic stress disorder (PTSD): a meta-analysis. Eur J Psychotraumatol 2019; 10: 1654782. 31489138 10.1080/20008198.2019.1654782 PMC6713125

[12] Ahmadpour N , Randall H , Choksi H , Gao A , Vaughan C , Poronnik P. Virtual reality interventions for acute and chronic pain management. Int J Biochem Cell Biol 2019; 114: 105568. 31306747 10.1016/j.biocel.2019.105568

[13] Feng H , Li C , Liu J , Wang L , Ma J , Li G et al. Virtual reality rehabilitation versus conventional physical therapy for improving balance and gait in Parkinson’s disease patients: A randomized controlled trial. Med Sci Monit 5; 25: 4186–4192. 10.12659/MSM.916455 PMC6563647 31165721

[14] Arane K , Behboudi A , Goldman RD. Virtual reality for pain and anxiety management in children. Can Fam Physician 2017; 63: 932–934. 29237632 PMC5729140

[15] Pallavicini F , Argenton L , Toniazzi N , Aceti L , Mantovani F. Virtual reality applications for stress management training in the military. Aerosp Med Hum Perform 2016; 87: 1021–1030. 28323588 10.3357/AMHP.4596.2016

[16] Yamane T. Statistics, An Introductory Analysis, 2nd Ed., New York: Harper and Row. 1967.

[17] Wolmeister AS , Schiavo CL , Nazário KCK , Castro SMJ , de Souza A , Caetani RP et al. The Brief Measure of Emotional Preoperative Stress (B-MEPS) as a new predictive tool for postoperative pain: A prospective observational cohort study. PLoS One 2020; 15: e0227441. 31914146 10.1371/journal.pone.0227441 PMC6948814

[18] Cao X , Yumul R , Elvir Lazo OL , Friedman J , Durra O , Zhang X et al. A novel visual facial anxiety scale for assessing preoperative anxiety. PLoS One 2017; 12: e0171233. 28196099 10.1371/journal.pone.0171233 PMC5308844

[19] Martin CR , Hollins Martin C , Redshaw M. The Birth Satisfaction Scale-Revised Indicator (BSS-RI). BMC Pregnancy Childbirth 2017; 17: 277. 28851307 10.1186/s12884-017-1459-5 PMC5575858

[20] Turan AZ , Yilmaz M , Saracoglu T. The effect of virtual reality glasses on anxiety during surgery under spinal anesthesia: a randomized controlled study. Anaesth Pain Intensive Care 2021; 25: 170–175.

[21] Frey DP , Bauer ME , Bell CL , Low LK , Hassett AL , Cassidy RB et al. Virtual reality analgesia in labor: The VRAIL pilot study-a preliminary randomized controlled trial suggesting benefit of immersive virtual reality analgesia in unmedicated laboring women. Anesth Analg 2019; 128: e93–e96. 31094789 10.1213/ANE.0000000000003649

[22] Ioannou A , Papastavrou E , Avraamides MN , Charalambous A. Virtual reality and symptoms management of anxiety, depression, fatigue, and pain: a systematic review. SAGE Open Nurs 2020; 6: 2377960820936163. 33415290 10.1177/2377960820936163 PMC7774450

[23] Piskorz J , Czub M. Effectiveness of a virtual reality intervention to minimize pediatric stress and pain intensity during venipuncture. J Spec Pediatr Nurs 2018; 23: 1. 10.1111/jspn.12201 29155488

[24] Gold J , Mahrer N. Is virtual reality ready for prime time in the medical space? a randomized control trial of pediatric virtual reality for acute procedural pain management. J Pediatr Psychol 2018; 43: 266–275. 29053848 10.1093/jpepsy/jsx129

[25] Aminabadi A , Erfanparast L , Sohrabi A , Naghili A. The impact of virtual reality distraction on pain and anxiety during dental treatment in 4-6-year-old children: a randomized controlled clinical trial. J Dent Res Dent Clin Dent Prospects. 2011; 6: 117–124. 10.5681/joddd.2012.025 PMC3529924 23277857

[26] Shetty V , Suresh LR , Hegde AM. Effect of virtual reality distraction on pain and anxiety during dental treatment in 5 to 8 year old children. J Clin Pediatr Dent 2019; 43: 97–102. 30730798 10.17796/1053-4625-43.2.5

[27] Gómez-Polo C , Vilches AA , Ribas D , Castaño-Séiquer A , Montero J. Behaviour and anxiety management of paediatric dental patients through virtual reality: A randomized clinical Trial. J Clin Med 2021; 10: 3019. 34300185 10.3390/jcm10143019 PMC8304330

[28] López-Valverde N , Muriel-Fernández J , López-Valverde A , Valero-Juan LF , Ramírez JM , Flores-Fraile J et al. Use of virtual reality for the management of anxiety and pain in dental treatments: systematic review and meta-analysis. J Clin Med 2020; 9: 3086. 32731319 10.3390/jcm9082404 PMC7464311

[29] Maples-Keller JL , Bunnell BE , Kim SJ , Rothbaum BO. The use of virtual reality technology in the treatment of anxiety and other psychiatric disorders. Harv Rev Psychiatry 2017; 25: 103–113. 28475502 10.1097/HRP.0000000000000138 PMC5421394

[30] Meyerbröker K , Morina N. The use of virtual reality in assessment and treatment of anxiety and related disorders. Clin Psychol Psychother 2021; 28: 466–476. 34097318 10.1002/cpp.2623 PMC8362145

[31] Walker MR , Kallingal GJ , Musser JE , Folen R , Stetz MC , Clark JY. Treatment efficacy of virtual reality distraction in the reduction of pain and anxiety during cystoscopy. Mil Med 2014; 179: 891–896. 25102532 10.7205/MILMED-D-13-00343

[32] Glennon C , McElroy S , Connelly L , Mische Lawson L , Bretches A , Gard A et al. Use of virtual reality to distract from pain and anxiety. Oncol Nurs Forum 2018; 45: 545–552. 29947355 10.1188/18.ONF.545-552

[33] Alaterre C , Duceau B , Sung Tsai E , Zriouel S , Bonnet F , Lescot T et al. Virtual reality for peripheral regional anesthesia (VR-PERLA Study). J Clin Med 2020; 9: 215. 31941129 10.3390/jcm9010215 PMC7019894

[34] Dumoulin S , Bouchard S , Ellis J , Lavoie KL , Vézina MP , Charbonneau P et al. Randomized controlled trial on the use of virtual reality for needle-related procedures in children and adolescents in the emergency department. Games Health J 2019; 8: 285–293. 31135178 10.1089/g4h.2018.0111

[35] Tharion JG , Kale S. Patient satisfaction through an immersive experience using a mobile phone-based head-mounted display during arthroscopic knee surgery under spinal anesthesia: a randomized clinical trial. Anesth Analg 2021; 133: 940–948. 34283040 10.1213/ANE.0000000000005666

[36] Baytar Ç , Bollucuoğlu K: Effect of virtual reality on preoperative anxiety in patients undergoing septorhinoplasty. Braz J Anesthesiol 2021: S0104-0014(21)00342-0. 10.1016/j.bjane.2021.08.014 PMC10068547 34562488

[37] Sahin G , Basak T. The effects of intraoperative progressive muscle relaxation and virtual reality application on anxiety, vital signs, and satisfaction: a randomized controlled trial. J Perianesth Nurs 2020; 35: 269–276. 32146074 10.1016/j.jopan.2019.11.002

[38] Hua Y , Qiu R , Yao WY , Zhang Q , Chen XL. The effect of virtual reality distraction on pain relief during dressing changes in children with chronic wounds on lower limbs. Pain Manag Nurs 2015; 16: 685–91. 25972074 10.1016/j.pmn.2015.03.001

[39] Massoth C , Töpel L , Wenk M. Hypotension after spinal anesthesia for cesarean section: how to approach the iatrogenic sympathectomy. Curr Opin Anaesthesiol 2020; 33: 291–298. 32371631 10.1097/ACO.0000000000000848

